# Conformable Holographic Metasurfaces

**DOI:** 10.1038/s41598-017-04482-2

**Published:** 2017-07-03

**Authors:** James Burch, Dandan Wen, Xianzhong Chen, Andrea Di Falco

**Affiliations:** 1 University of St Andrews, School of Physics and Astronomy, St Andrews, KY16 9SS UK; 2 Heriot-Watt University, Institute of Photonics and Quantum Sciences, Edinburgh, EH14 4AS UK

## Abstract

Metasurface holograms are typically fabricated on rigid substrates. Here we experimentally demonstrate broadband, flexible, conformable, helicity multiplexed metasurface holograms operating in the visible range, offering increased potential for real life out-of-the-lab applications. Two symmetrically distributed holographic images are obtained when circularly polarized light impinges on the reflective-type metasurface positioned on non-planar targets. The two off-axis images with high fidelity are interchangeable by controlling the helicity of incident light. Our metasurface features the arrangement of spatially varying gold nanorods on a flexible, conformable epoxy resist membrane to realize a Pancharatnam-Berry phase profile. These results pave the way to practical applications including polarization manipulation, beam steering, novel lenses, and holographic displays.

## Introduction

Metasurfaces (MSs) are ultra-thin artificial materials made of individual structures, called meta-atoms. These meta-atoms dictate the optical properties of the resulting metamaterial with their specific shape, size, orientation, and arrangement. MSs are essentially planar and thus simpler to fabricate than bulk MMs^1^, with significant consequences for practical applications. One of the prominent features of MSs is that they permit to control the phase and amplitude of impinging light over scales much smaller than its wavelength^1, 2^. Thus, it is possible to design MSs with extremely complex behavior. Some recent examples include the generalization of Snell’s law^3^, flat lensing^2, 4^, ultra-broadband coherent perfect absorption^5^, beam steering^6, 7^, ultrathin vortex wave plates for applications in optical tweezers and optical communication systems^8^, and wide angle filters^9^. Additionally, the optical response of MSs can be tuned, by varying geometrical or physical parameters, e.g. for applications in polarization manipulation^10^, tunable absorption^11, 12^, laser steering^6, 7^, signal modulation^13^, nonreciprocal EIT^14^, and time-varying MS and Lorentz nonreciprocity^15^. The capability to engineer the phase of an optical beam with high accuracy and spatial resolution is perfectly suited for holographic applications. This has been exploited to demonstrate several applications in security^16^, displays^17^ and the storage and manipulation of information^18, 19^. MSs allow for pixel by pixel tailoring of the phase profile of the hologram, allowing for highly efficient designs compared to amplitude only holograms^20, 21^. Holographic MSs use the 2D arrangement of meta-atoms to produce an image from the scattered incident light. Such an image can be computer generated by means of iterative phase reconstruction algorithms, such as the Gerchberg-Saxton^22^. Furthermore, these images can embed and exploit novel degrees of freedom, such as the helicity^16^, or polarization of the incident light^23, 24^, with profound implications e.g. for the analysis of DNA structure^25^ and stereochemistry^26^. Realizing holographic metasurfaces on flexible substrates brings about several advantages. Firstly, flexible MSs are compatible with roll-to-roll production and printed electronics^27^. Roll-to-roll production is of great interest for bringing MSs inexpensively, and in high volume, to market, given its relative technological maturity^28–30^.

Additionally, flexible MSs can also be mechanical tuned through stretching or vibration^1, 31, 32^, e.g. to vary the focal length of a metasurface lens^33^. Lastly and most importantly, flexible MS are conformable to non-planar surfaces, which allows for novel and diverse applications aimed at providing traditional, and dull materials with an advanced photonic layer, e.g. to include ultrathin devices on, clothing, optical fibers^34, 35^, contact lenses^36^, packaging and bio-inspired applications^37^. In this paper we designed, fabricated and characterized flexible holographic MSs to project a holographic image. Our holographic images were characterized before and after the lift-off process from a substrate, and placed on a substrate which could not be nanopatterned directly and that is non-perfectly planar. This allowed us to observe the conformability of our MS to different substrates. Our holographic MS works in reflection for incident light, and two image designs were tested with helical polarizations.

## Results

### Hologram design

**Figure 1:**
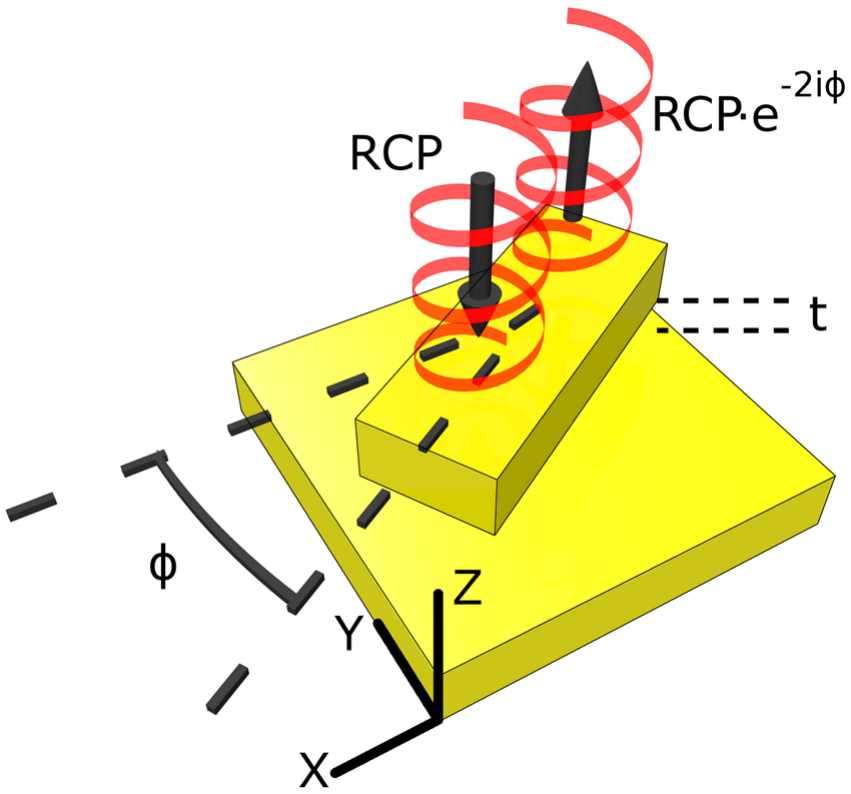
A diagram of the unit cell structure of a nanorod with incident right hand circularly polarized light. The spacing between the nanorod and backplane is denoted as *t*. The angle *ϕ* belongs to the XY plane and is defined between the long axis of the nanorod and X axis.

One of the most successful schemes to realize a broadband, broad angle, holographic MS is the reflective geometry as shown in Fig. 1. These devices typically consist of three layers, a nano-patterned surface containing the meta-atoms, a spacing layer, and a reflective backplane. The three-layer design acts as a Fabry-Perot cavity to boost phase conversion efficiency, and reduce the dispersion typical of a single MS layer^16, 21^. Using this approach, researchers have achieved efficiencies of 60–80%^16, 21^.

The meta-atoms are metallic nanorods, with plasmonic resonances characterized by a fast and slow axis, due to the form factor. By tailoring the exact dimensions of the nanorod to obtain a phase change of *π* between these axes, the nanorod emulates a reflective type half waveplate^21^. The reflected beam has the same handedness of the incident one, but with an additional phase of 2 *ϕ* ^21^. Such a half waveplate can thus be used to accurately control the phase of reflected light over a broad wavelength range. The unconverted light is reflected back in the 0th order beam. We used the Gerchberg-Saxton algorithm to iteratively retrieve the phase profile to generate the required far field image. Helicity multiplexed holograms differ from conventional linearly polarized holograms in that the two centrosymmetric images can be exchanged based on the helical direction of incident light. This exchange occurs because the intensity distribution in the far field is dependent on the sign of the phase function of the incident light^16^. An off-axis holographic design was chosen to separate the images from the zeroth order beam. To iteratively retrieve the phase, we assumed a uniform planar source wave, and that there exists a Fourier transform relation between the hologram plane, and the far field. We then encode the phase profile in the hologram plane onto the MS by the angle of gold nanorods point by point. The retrieved phase profile is realized by 1000 × 1000 nanorods with a neighboring distance of 300 nm, and an area of 0.3 mm × 0.3 mm. To reduce the size of diffraction spots and improve image quality in the far field we constructed a 2 × 2 periodic array of our retrieved phase profile. As such our MS had a total area of 0.6 mm × 0.6 mm. The nanorod spacing was chosen to create an image with a projection range of 95° at *λ* = 650 nm, following the simple geometric relationship

$${\rm{\Delta }}P={d}_{i}/{M}_{i}={m}_{i}\lambda /({M}_{i}\times 2{\rm{t}}{\rm{a}}{\rm{n}}(\frac{{\alpha }_{i}}{2}))$$

where Δ*P* is the nanorod spacing, *d*_*i*_ is the period of the 2 × 2 array, *m*_*i*_ and *M*_*i*_ are the number of pixels in the far field and in the MS respectively, *λ* is the wavelength of the light, *α*_*i*_ is the angular range, and *i* = *x*, *y*, as detailed in^21^. The spacing between the nanorods must be small enough to satisfy the Nyquist-Shannon sampling theorem for a continuous phase profile, whilst keeping the distances large enough to minimize cross-talk between the nanorods. To further limit cross-talk between nanorods we used 16 discrete nanorod angles. The angle of 22.5° was chosen to maximize the minimum distance between nanorod corners, whilst keeping frequent sampling across all angles. Each nanorod angle encodes twice the retardation in the Pancharatnam-Berry phase angle of light transmitted through the nanorod.

### MS fabrication

**Figure 2:**
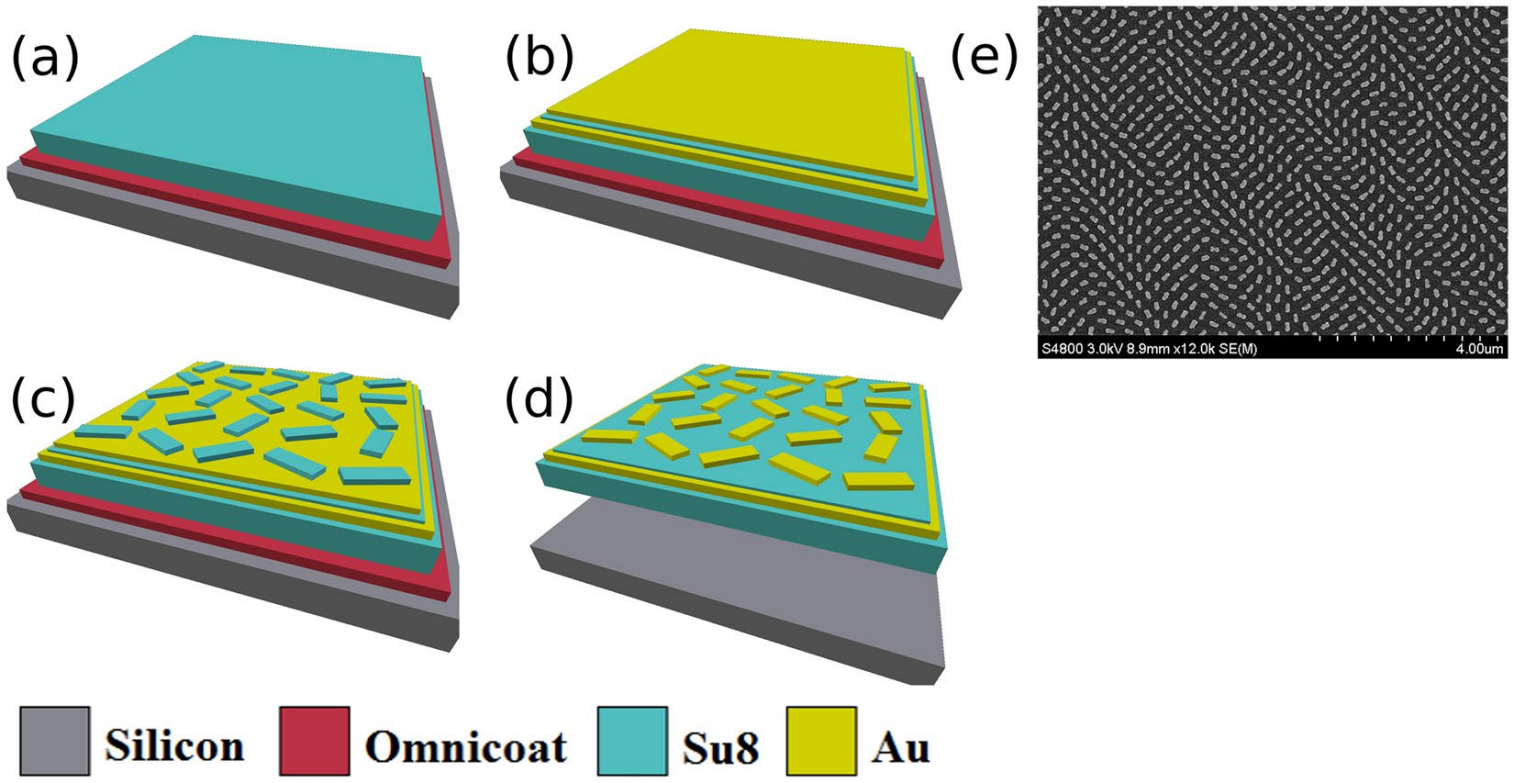
Schematic of the fabrication process and the fabricated flexible MS hologram. (**a**) A silicon substrate is initially coated with an Omnicoat layer and a thick SU-8 film by spin-coating. (**b**) A gold film is evaporated onto the SU-8 film as a reflective backplane. After that, a spacing layer of SU-8 is then spun onto the gold film. Finally, a second gold film is deposited on the SU-8 film. (**c**) A resist layer of SU-8 is spun onto the final gold layer for the standard electron beam lithography process. Nanorods are defined after the development process and then used as the etching mask. (**d**) Gold nanorods on the top of the sample are then obtained after a reactive ion etch. The Omnicoat layer is then dissolved to leave a free-floating flexible hologram. (**e**) An SEM image of a typical area of the nanorod MS taken after lift-off. It was observed that there were no discernible visual differences between the SEM images of the sample before, and after lift-off (see Supplementary Fig. S1).

To fabricate the flexible MS hologram we followed the procedure sketched in Fig. 2, and outlined in detail in the methods section.

Firstly we spin coated, on a silicon carrier, the lift off layer. Secondly we spin coated a thick polymer layer to be used as the MS substrate once lift-off has occurred. We then evaporated a gold film onto the sample to act as a reflective backplane. Next we spin coated a thin polymer layer to be used as a spacer between the gold backplane and the MS. We then deposited a thin layer of gold on the sample and spun photoresist on top. After a standard electron beam lithography process, we used a reactive ion back etch to define gold nanorods on top of our spacing layer. The lift-off layer could then be dissolved to leave a free-floating membrane. After lift-off we floated the sample on deionized water, and transferred it onto the desired substrate, for further characterization. As detailed below, in particular we placed the MS on a flat silicon substrate, and on the curved lens of a pair of safety glasses. Figure 2(e) displays an image of a typical region of the MS after lift-off taken by a scanning electron microscope, which shows that the quality of the nanopattern is not affected by the lift-off procedure.

### Experimental setup

**Figure 3:**
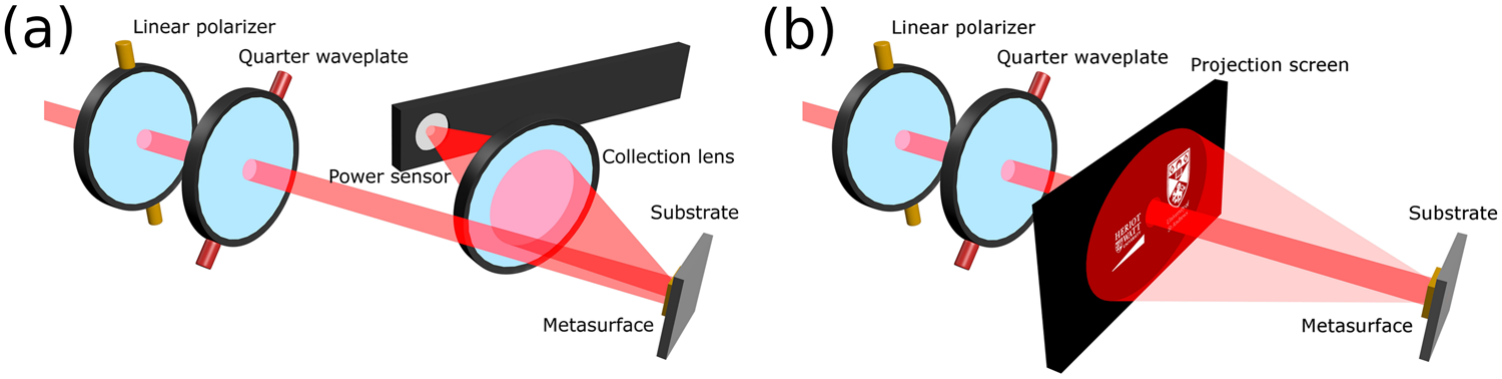
Schematics of the optical set-ups. The helicity of incident light on the MS is controlled by the relative angle between the linear polarizer and the quarter waveplate. In these images right handed circularly polarized light is shown. (**a**) To make efficiency measurements the light scattered by the MS is collected by a f = 25.4 mm lens and the intensity is measured with a Thorlabs S130C Photodiode Power Sensor (**b**) To photograph the holographic image the light scattered from the MS is projected onto a screen.

To quantitatively assess the viability of the process, we characterized the efficiency of the holographic MS before, and after lift-off from the rigid silicon substrate. The MS was excited with a SuperK EXTREME supercontinuum laser in the range 570–850 nm, in increments of 20 nm, with the setup sketched in Fig. 3(a) and (b). By passing the laser through a linear polarizer, and a quarter waveplate, we created light with polarizations from linear to circular with the required helicity. Because the unconverted light remains in the 0th order, and our image is centrosymmetric, we didn’t need to use additional polarizers after reflection from the sample.

To calculate the optical efficiency of our device we collected the reflected power on one side of the hologram, focusing the image with a lens of f = 25.4 mm on a Thorlabs S130C Photodiode Power Sensor, as shown in Fig. 3(a). This value was normalized to the incident power, measured by placing the lens and detector directly in front of the quarter waveplate. Since the beam size was larger than the patterned area, we scaled the efficiency by the ratio of the power incident on the MS and the total incident power. The beam size as function of wavelength was measured using a Thorlabs BC106N-VIS/M CCD beam profiler and the correction factor is shown in Supplementary Fig. S2. The total efficiency of the hologram was calculated considering the combined two sides of the holographic image. This was done by doubling the efficiency from one side of the holographic image. The efficiency vs wavelength measurements were made in the range 570–850 nm at normal incidence. Efficiency vs angle measurements were made at 650 nm. For photographing the holographic images, we projected the light incident on the MS onto a paper screen 100 mm from the MS as in Fig. 3(b). We then photographed the screen with a standard camera. The hole in the screen had a diameter of 3 mm.

### Helicity multiplexed holograms

**Figure 4:**
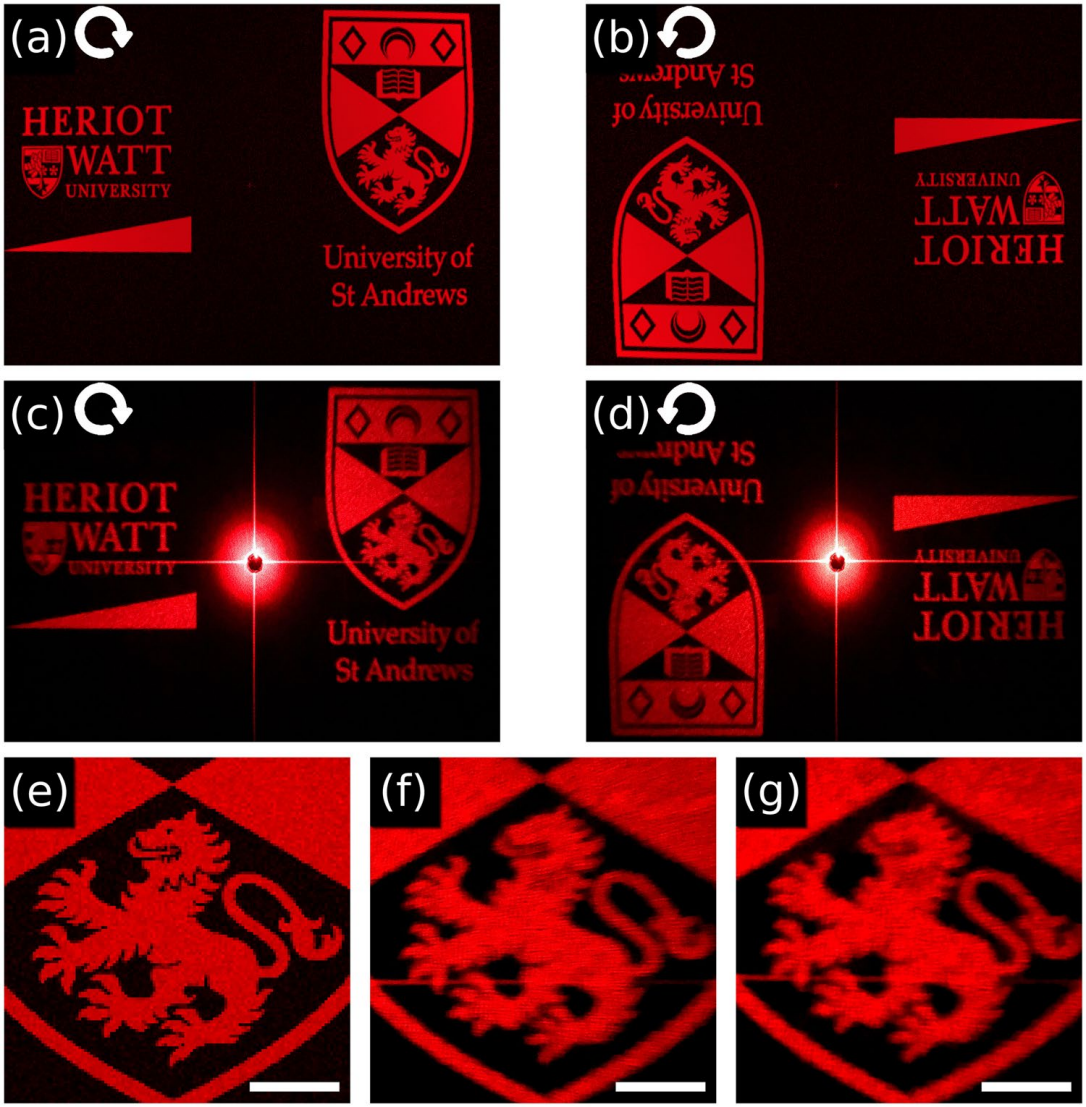
Reconstructed images for the incident light with a wavelength of 650 nm at normal incidence. The simulation results for the incident light with (**a**) right circular polarization and (**b**) left circular polarization and the corresponding experimental results after lift-off shown in (**c**) and (**d**). (**e**) Simulated close up of the lion. The comparison of experimentally measured close up of the lion (**f**) before and (**g**) after lift-off from the substrate. The scale bar is 10 mm with a MS to screen spacing of 100 mm. The University of St Andrews logo is © University of St Andrews and used with permission. The Heriot-Watt logo is © Heriot-Watt University and used with permission.

Figure 4 shows that our flexible device can form high fidelity helicity multiplexed holograms. Figure 4(a) and (b) show the numerically generated target images for right and left handed circular polarizations respectively. The experimental images 4(c)and (d) were acquired using the setup of Fig. 3(b). The distortion due to the spherical far field being projected onto a flat plane has been pre-compensated for as detailed in Supplementary Fig. S3. In the far field the focus of our holographic images is robust to the exact distance to the screen, and their size scales linearly with this distance without distortion. This occurs because the image is designed to be viewed at infinity, so once the image is formed there are no further distortions as distance from the MS increases. The cross in the center of Fig. 4(c) and (d) is a simple measurements artifact, due to the MS acting as a square aperture. The movie in the SI shows the dynamic transition between the two polarization states. Panels (e–g) of Fig. 4 show a zoomed in view of the designed image, and of the images obtained before and after lift-off, respectively. These results demonstrate that the process of lift off does not lead to any visible degradation in the image fidelity and signal to noise ratio. Furthermore these results show that our process is compatible with holograms that are both high fidelity, and containing high frequency terms in the visible range.

**Figure 5:**
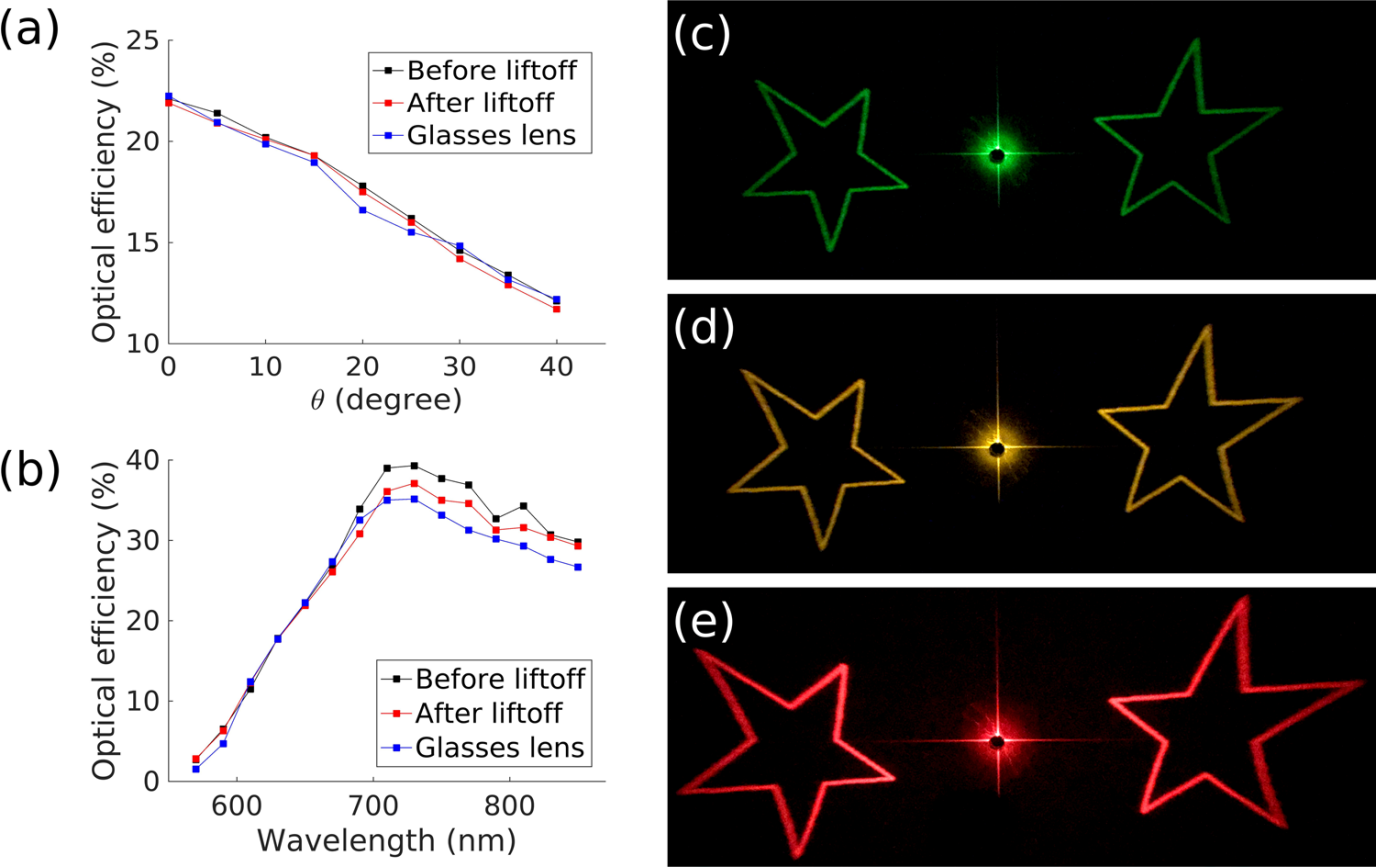
Measured efficiency for right handed incident circular polarization vs (**a**) the angle between the normal to the surface of the MS and the incident beam for *λ* = 650 nm and (**b**) the wavelength of the beam for normal incidence. Experimentally obtained images after lift-off for the light beams at (**c**) 570 nm, (**d**) 590 nm, and (**e**) 690 nm. These images were taken with right handed circular polarization at normal incidence.

**Figure 6:**
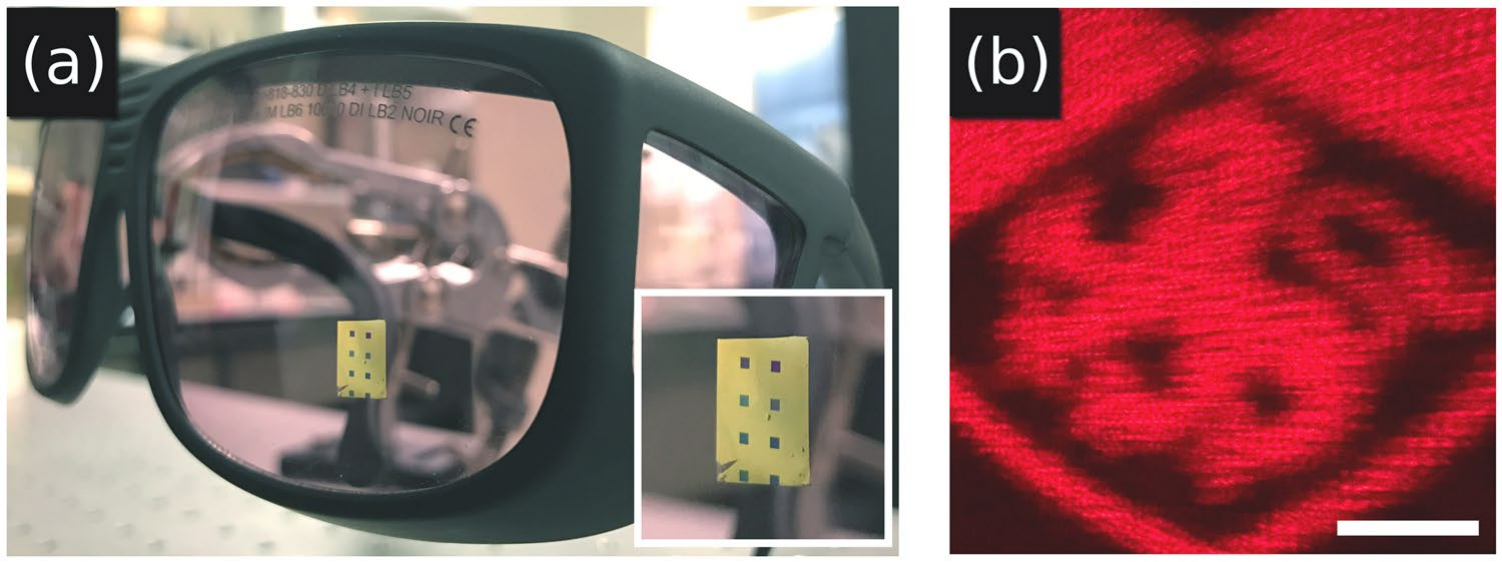
(**a**) Our MS conformed to a pair of safety glasses. The inset is a close up of the MS. (**b**) An experimentally obtained image with the MS conformed to the glasses for the incident light with a wavelength of 650 nm at normal incidence. The scale bar is 10 mm with a MS to screen spacing of 100 mm. The University of St Andrews logo is © University of St Andrews and used with permission. The Heriot-Watt logo is © Heriot-Watt University and used with permission.

In Fig. 5 we measured performance characteristics of our holographic MS to compare the efficiency and angular robustness of the MS before and after lift-off, when placed on a flat silicon substrate and on a curved glasses lens as in Fig. 6. We observed that the efficiency of our design decreased roughly linearly with increasing angle as can be seen in Fig. 5(a). In Fig. 5(b) we show that for right hand circularly polarized light, and a 99% dark image, our device peaks in efficiency around 730 nm at 40% before lift-off, 37% after lift-off, and 35% after adhering to the glasses lens. As such neither the lift-off process, nor the adhesion to a glasses lens significantly impacts the holographic efficiency. At wavelengths far from 730 nm the dimensions of the nanorods, and the thickness of the spacing layer, are not optimized to produce the *π* phase change and so this efficiency decreases. Furthermore, at shorter wavelengths the interband transitions in gold cause excessive absorption. This reduces the efficiency of the device to the point that holographic images cannot be seen much below 570 nm. Despite this limitation we decided to use gold as the plasmonic material, because unlike silver, it can be back-etched with a reactive ion etch. This was important as RIE defines nanoscale features better than a lift-off procedure on our SU-8 polymer spacing layer. For applications requiring the whole visible range gold could be interchanged with silver^16^, or aluminum^38^, if chlorine is used during the etch. Furthermore, gold is strongly inert which leads to better stability of nano-sized gold features than silver or aluminum. Figure 5(c–e) were captured in the visible range, after lift-off, to visually display the efficiency at different wavelengths, and the centrosymmetric pattern that we used in the efficiency readings.

To demonstrate that the flexible membranes retain their functionality when transferred to different targets, as visible in Fig. 6, we placed our MS on a glasses lens with a radius of curvature of 140 mm. This corresponds to a phase change of 1.1 *π* between the outside and center of the MS. Qualitatively it can be seen in Fig. 6(b) that there is some defocussing effect, and a lower signal to noise ratio than Figs. 4(f) and (g). Clearly the effect of curvature can be detrimental to the image quality, but this could also offer opportunities to be investigated in other studies.

A strength of our approach is that we can tailor devices for specific use cases. Here the MS conforms to the general shape of the object it rests on, in this case a glasses lens. The impact of fine surface roughness can be negated by adjusting the thickness of the manipulation layer. A thicker manipulation layer will conform less to the surface roughness and is thus ideal for holography. However, a thinner manipulation layer could be used when a higher degree of conformability to the surface is required. This would be the case for tightly curved surfaces.

## Discussion

We have designed and fabricated a flexible, conformable, holographic MS capable of supporting helicity multiplexed holograms in the visible range. Our MS is realized with gold nanorods positioned point by point to define a Pancharatnam-Berry phase profile. By using a three-layer reflective design on our flexible manipulation layer we can obtain both high efficiency, and conformability to planar and curved surfaces. By then controlling the helicity of the incident light the two high fidelity, broad angle, broadband, images can be interchanged at will. Such a device is of practical relevance for tunable MS devices with applications including polarization manipulation, beam steering, novel lenses, and holographic displays. Due to the conformability of our device these technologies could be realized on a target that can not be otherwise functionalized with photonic nanopatterns. Additionally, the flexibility of this device opens up the possibility of roll-to-roll printing to vastly decrease the cost, and increase the throughput, of MS production.

## Methods

### Fabrication

We first spin coated, on a silicon carrier, the lift off layer (Omnicoat, from Microchem), at a speed of 1000 rpm for 1 minute, followed by 1 minute of baking at 230 °C. Then we deposited a thick polymer layer to be used as the MS substrate. For this purpose, we chose SU-8, a negative-tone, epoxy based resist from Microchem (MS USA), and available in different formulations. We found that using a blend of SU-8 2050 and SU-8 2000.5, mixed 1:1 and spun at 5000 rpm gives a thickness of 2.6 *μ*m. This thickness was chosen to guarantee both high flexibility, and robustness of the MS. We exposed this layer to UV light for 5 minutes and completed the cross-linking process by heating the substrate to 100 °C for 5 minutes. We then deposited, via electron beam evaporation, a 100 nm thick layer of gold, enough to guarantee the efficient reflection of light. Next we used SU-8 to realize the spacing layer, by blending SU-8 2000.5 and Cyclopentanone in a 1:3 ratio, and spinning this mixture at 6000 rpm to a thickness of 90 nm. We also exposed this layer to UV light before curing for 2 minutes at 100 °C to cross-link the polymer. We characterized the optical dispersion properties of this film via a standard retrieval method^39^, which gave a refractive index of 1.67 at *λ* = 600 nm. The variation in refractive index was less than 1.5% in the wavelength range 570–850 nm. The top layer of gold for the meta-atoms was 40 nm thick. The meta-atoms were written with a Raith eLINE Plus electron beam device at 30 kV, with a dose of 5 *μ* C/cm^2^, on a photoresist made of a 1:2 blend of SU-8 2000.5 and Cyclopentanone, spun at 5000 rpm and baked for 5 minutes at 90 °C. Ethyl lactate was used to develop the SU-8 resist after a post-exposure baking step of 2 minutes at 100 °C. An Ar based reactive ion back-etch was then used to etch the gold, leaving nanorods with the dimensions 75 nm × 200 nm × 40 nm. The parameters of this etch were a DC bias of −325 V, and a pressure of 0.05 mBar. The sample was then hard baked at 150 °C for 5 minutes to slightly increase the rigidity of the supporting membrane, to simplify the release step. To lift-off the MS from the silicon carrier, we used Microposit MF319 to dissolve the Omnicoat layer.

## Electronic supplementary material

Supplementary Information

Supplementary movie
