# Supplementary material for: Conformable Holographic Metasurfaces
Source: Sci Rep. 2017 Jul 3;7:4520. doi: 10.1038/s41598-017-04482-2 (PMC5495780; doi:10.1038/s41598-017-04482-2)
Supplement: Supplementary file 1 — Supplementary Information [file 41598_2017_4482_MOESM1_ESM.pdf]

Supplementary Information Conformable Holographic Metasurfaces

James Burch[1,*], Dandan Wen[2], Xianzhong Chen[2], and Andrea Di Falco[1]

[1]University of St Andrews, School of Physics and Astronomy, St Andrews, KY16 9SS, UK
[2]Heriot-Watt University, Institute of Photonics and Quantum Sciences, Edinburgh, EH14 4AS, UK
*jb298@st-andrews.ac.uk, adf10@st-andrews.ac.uk

S1. Comparative SEM images of the MS before and after lift-off

We took SEM images of the MS before and after lift-off, to assess if the process would affect the quality of the nano-features. Fig. S1(a) displays an area of the MS before lift-off, and fig. S1(b) displays a different area of the same MS after lift-off. The quality of the MS is identical in both cases.

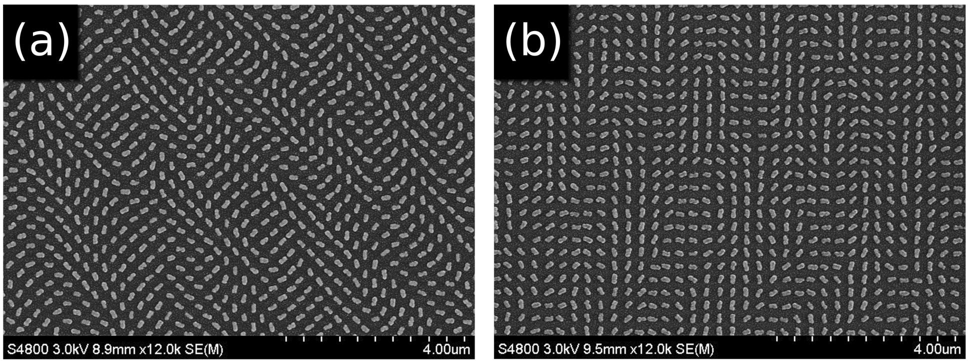

Figure S1 An SEM image of typical areas of the nanorod MS taken (a) before lift-off (b) after lift-off.

S2. Efficiency correction factor

Because the laser beam diameter was larger than the MS, we identified a calibration factor to find the power incident on the MS only. This calibration is a function of the wavelength and is shown in fig. S2. The calibration factor is defined by the ratio of the integral of the beam intensity over the area of the MS and the total intensity. We found this calibration using a Thorlabs BC106N-VIS/M CCD beam profiler to characterize the shape of the beam. Two calibration factors were made, one for the before and after lift-off cases, and one for the case where the MS was adhered to the glasses lens. Only the former is shown as the two are similar.

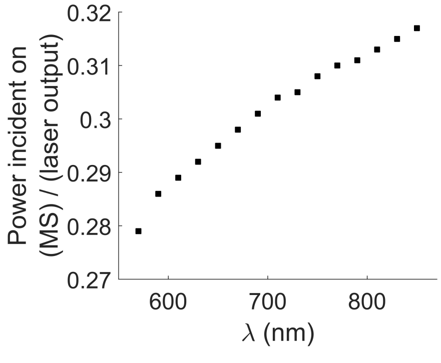

Figure S2 Experimental correction factor as function of the wavelength.

S3. Image pre-compensation

In creating our holographic phase profiles, we assume a spherical propagation into the far field. For photographing our images however, we use a planar screen. As such, without pre-compensation, the far field image is distorted. To pre-compensate for the distortion, we adjusted the target image from fig. S3(a) to fig. S3(b) using a standard spherize filter, similar to that used in popular photo editing software, programmed in Matlab. The radius of curvature of this filter is chosen to equal that of the far field at the position of the screen, in our case 100 mm. This pre-compensation does effectively lower the resolution at the edges of the image of the target image however. This combined with the non-optimized camera focus contributes to the poorer fidelity of the edges of Fig. 4(c) and 4(d) of the main manuscript.

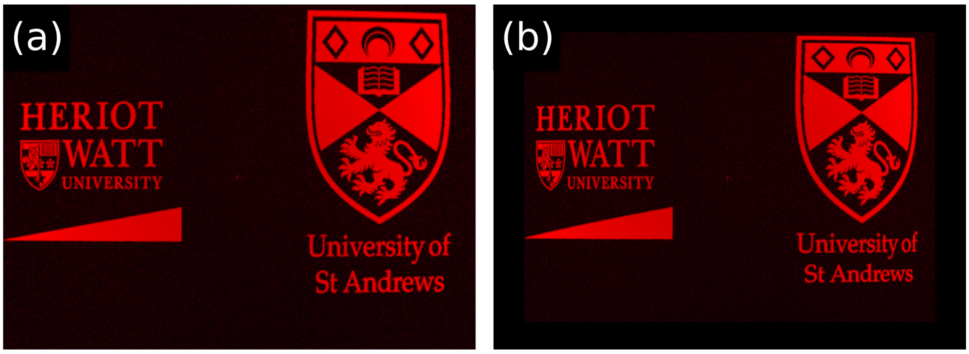

Figure S3 The holographic target image (a) without pre-compensation (b) with pre-compensation. The University of St Andrews logo is ©University of St Andrews and used with permission. The Heriot-Watt logo is ©Heriot-Watt University and used with permission.
